# Endovascular treatment of a tuberculous aneurysm of the pararenal abdominal aorta leading to an aortoduodenal fistula

**DOI:** 10.1016/j.jvscit.2022.07.011

**Published:** 2022-08-06

**Authors:** Shuhei Azuma, Shinji Fukuhara, Yoshii Yasuyoshi

**Affiliations:** Department of Cardiovascular Surgery, Kyoto Katsura Hospital, Kyoto, Japan

**Keywords:** Aortoduodenal fistula, Tuberculous aneurysm, EVAR

## Abstract

This case report describes the presentation of a 79-year-old woman with no significant past medical history diagnosed with a saccular aneurysm with an aortoduodenal fistula. An emergency endovascular aneurysm repair was performed. Although the postoperative course was uneventful, 10 months after endovascular aneurysm repair, the patient died of miliary tuberculosis from mycotic aneurysms. Mycotic aneurysms are uncommon, and mycotic aneurysms caused by *Mycobacterium tuberculosis* are even rarer. Therefore, we believe our study makes a significant contribution to the literature given the rarity of the condition and suggests the importance of maintaining a high index of suspicion for tuberculosis as a possible cause of aortoduodenal fistula in primary mycotic aneurysm.

## Case report

**Fig 1:**
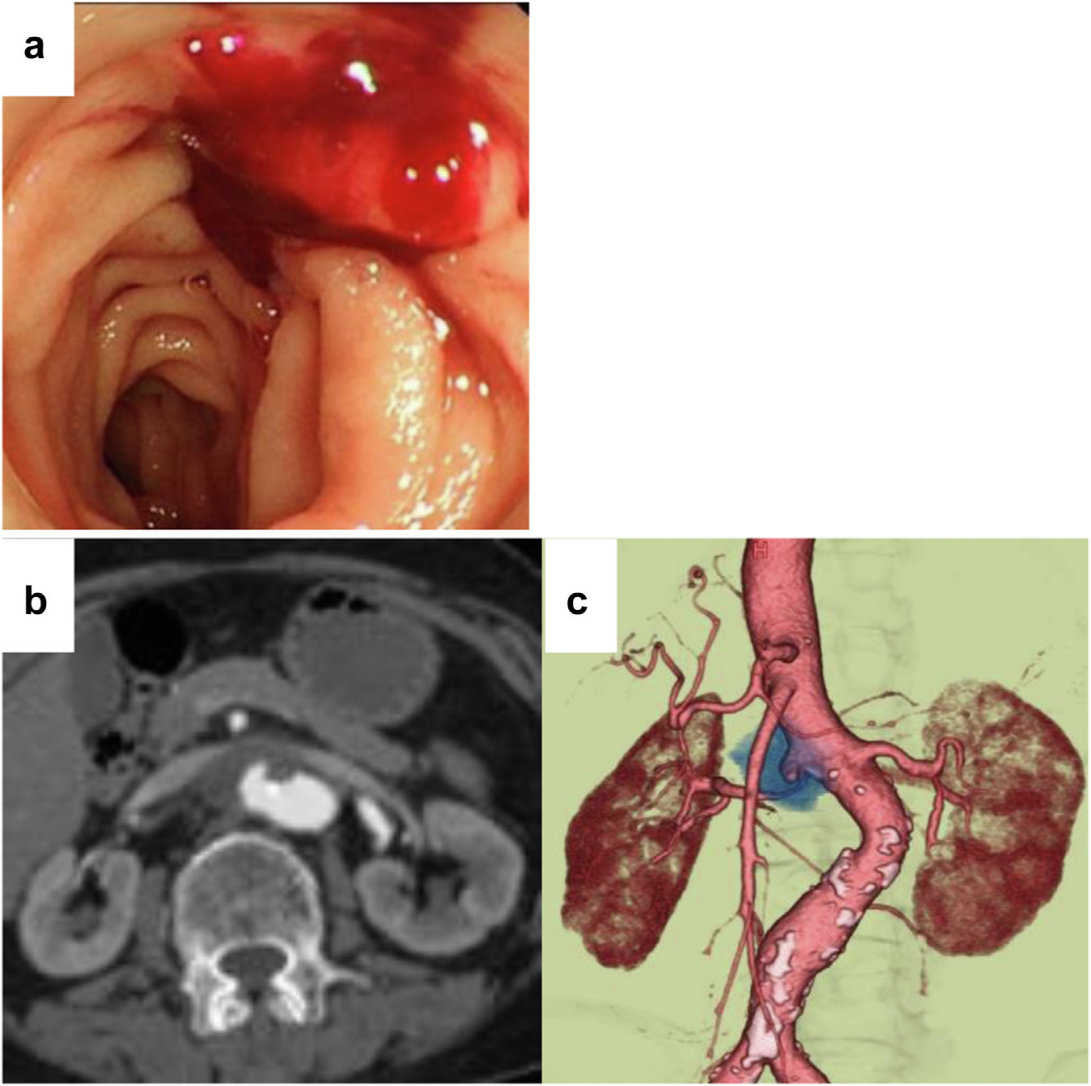
Preoperative enhanced abdominal computed tomography (CT) scan and endoscopy. **a,** Endoscopy demonstrated the fresh clot in the third portion of duodenum. There was no active bleeding. **b,** Axial view of the saccular aneurysm just above the right renal artery. **c,** Three-dimensional CT scan of the aneurysm oppressed the right renal artery.

A 79-year-old woman without a significant past medical history (including any previous history of infections) was admitted to our emergency department with massive melena and hemorrhagic shock. The patient was afebrile and suffered from severe hypotension (systemic blood pressure of 60 mm Hg) with an oxygen saturation of 98% on room air. Laboratory data showed a hemoglobin level of 6.0 g/dL; however, the remaining values were within normal limits. Chest radiography revealed no signs of pneumonia. A computed tomography (CT) scan and endoscopy were performed while systemic blood pressure was maintained at greater than 90 mm Hg by blood transfusion. Imaging revealed a pararenal abdominal aortic saccular aneurysm with an aortoduodenal fistula at the level of the third portion (Fig 1). An open procedure consisting of replacement of the thoracoabdominal aorta with graft and reconstruction of the visceral and renal vessels was entertained; however, owing to the hemodynamic instability and magnitude of an open procedure on this elderly patient, an emergent endovascular aneurysm repair (EVAR) with sandwich and chimney techniques under general anesthesia was chosen.

**Fig 2:**
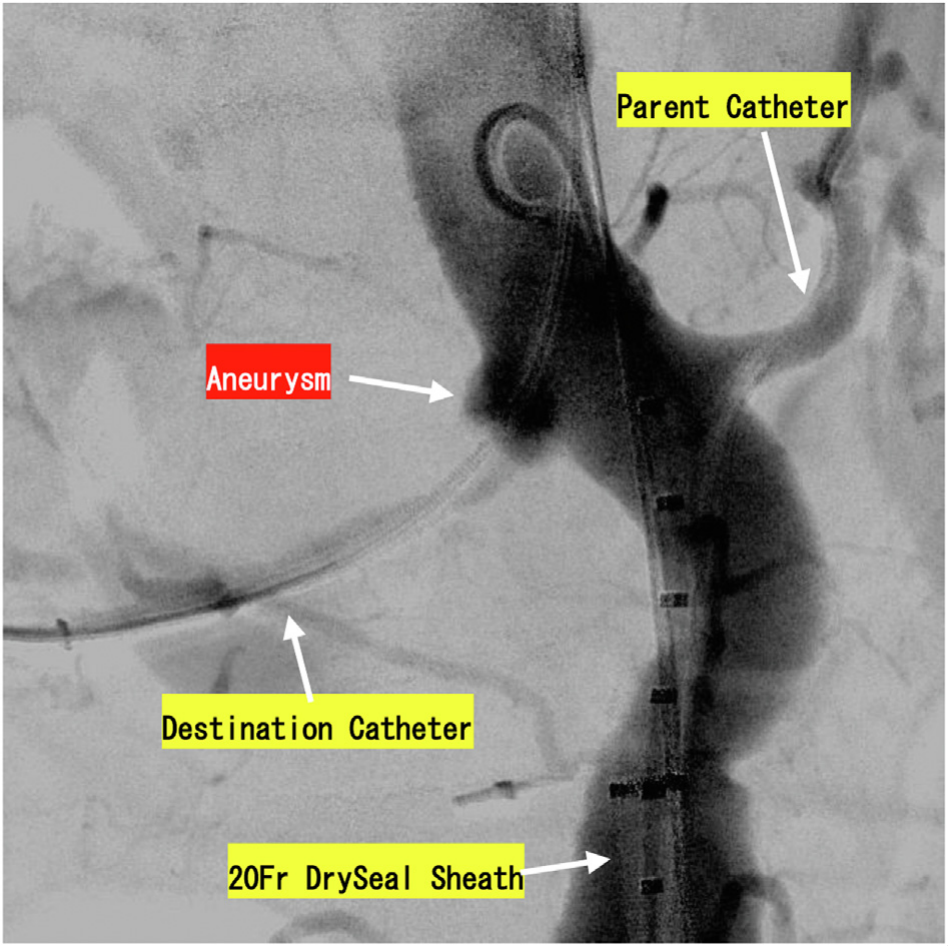
Intraoperative selective angiography. A 20F DrySealSheath was inserted in the left femoral artery. A 6F parent catheter and 6F destination catheter were inserted in the left renal artery and the right renal artery, respectively.

After bilateral femoral surgical access was obtained, a self-expanding covered stent, Viabahn (Gore Viabahn Endoprosthesis, W. L. Gore & Associates, Flagstaff, AZ), and a self-expanding bare-metal stent, SMART (Cordis Johnson & Johnson, Fremont, CA), were inserted into the right and left renal arteries, respectively, before the EVAR. The Gore Excluder Cuff endoprosthesis (W. L. Gore & Associates) was deployed just below the superior mesenteric artery, and the Medtronic Endurant cuff (Medtronic PLC, Minneapolis, MN) was added to extend the distal landing zone (Fig 2). The Endurant cuff with bare stents on the proximal edge may be difficult to remove during open surgery; therefore, we decided to place the Gore cuff without a bare stent first, followed by the Endurant cuff inside the Gore Cuff. Considering the length of the lesion, a longer Endurant cuff of 5 cm was used.

**Fig 3:**
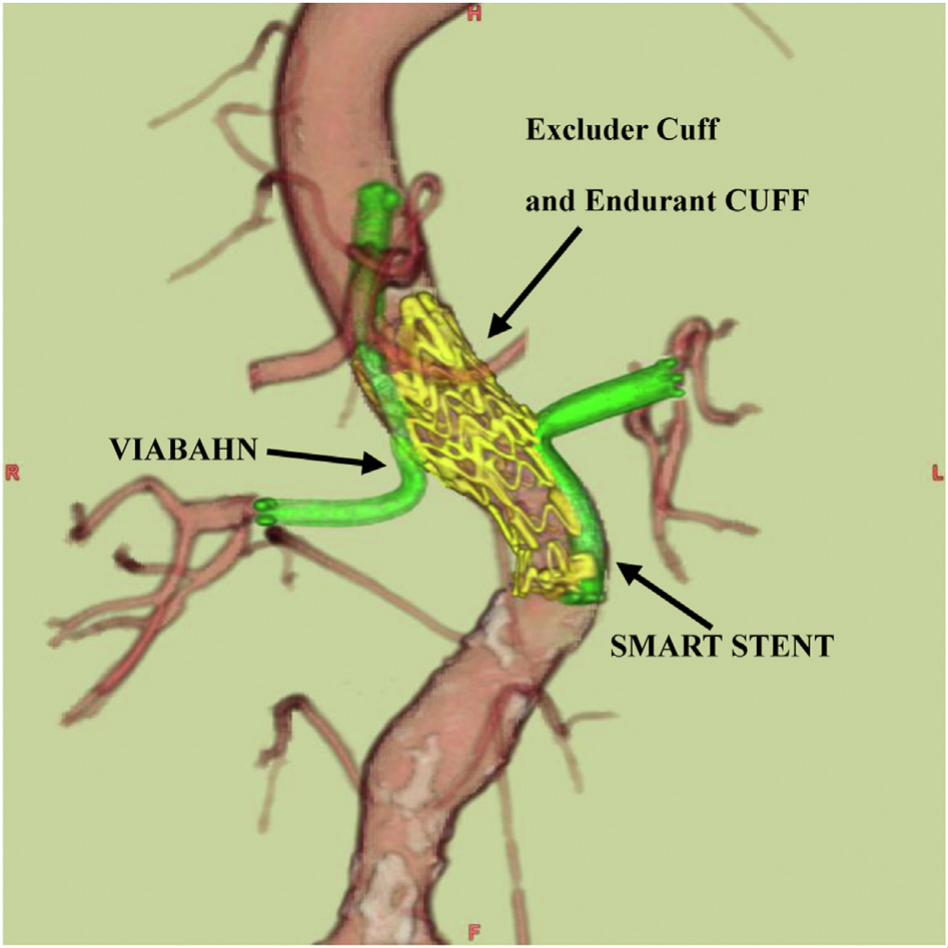
Postoperative enhanced three-dimensional computed tomography CT (3D-CT) scan. The 3D-CT scan showed that there was no endoleak and the bilateral renal artery were preserved. After the Viabahn stent was deployed in the right renal artery, the Excluder Cuff and Endurant Cuff were inserted just below the superior mesenteric artery. Finally, a SMART STENT was deployed in the left renal artery.

The procedure was successful, and a postoperative CT scan revealed exclusion of the aneurysm and no endoleak (Fig 3). Postoperatively, the progression of anemia owing to the gastrointestinal bleeding subsided. The patient had no further bleeding and her hemoglobin level improved. No pathogens were detected in the blood culture, and we did not send the blood cultures for tuberculosis testing. Considering the good postoperative course, the patient’s general condition, the lack of signs of infection, and the elderly patient’s ability to tolerate open surgery, we decided to follow-up the patient with a new oral quinolone antibiotic (Cravit 500 mg).

**Fig 4:**
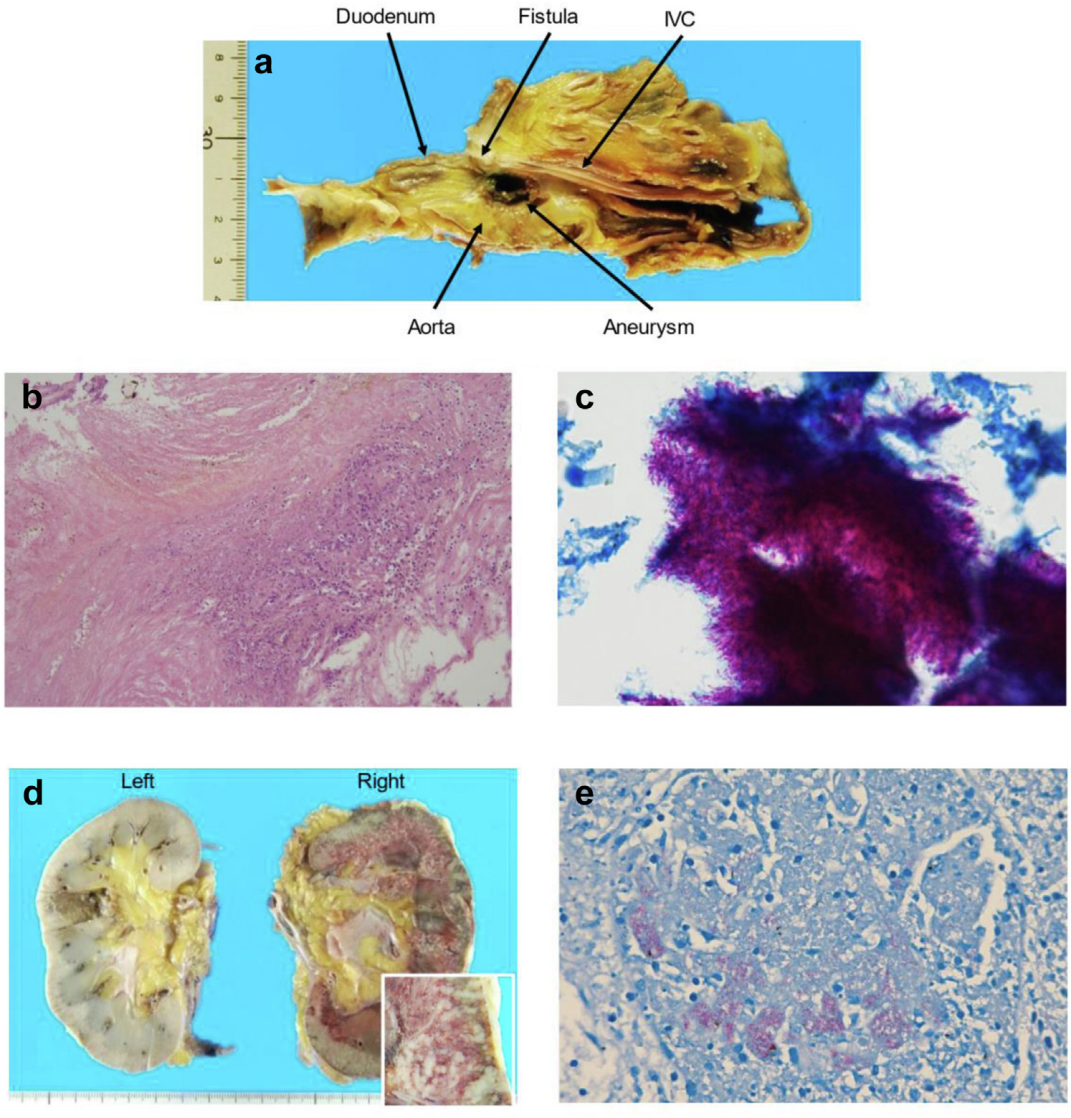
Pathological findings. **a,** Macroscopically, an aortoduodenal fistula is identified and an aortic aneurysm is located in the vicinity of the fistula. **b,** Hematoxylin and eosin staining shows necrotic tissue in the aneurysm (original magnification ×10). **c,** Ziehl-Neelsen staining shows numerous acid-fast bacilli in the necrotic tissue in the aneurysm (original magnification ×100). **d,** Macroscopic view of the kidneys shows renal involvement of military tuberculosis. The lesions are more conspicuous in the right kidney. The inset shows enlarged view of the right renal cortex. **e,** Ziehl-Neelsen staining shows numerous acid-fast bacilli in a glomerulus of the right renal cortex (original magnification ×40).

The patient was discharged on postoperative day 30 after the implantation without any signs of infection and was instructed to take antibiotic therapy for 12 months owing to the presence of a preoperative aortoduodenal fistula. A follow-up CT scan without contrast was performed at 1, 3, and 5 months after the operation, revealing a marked decrease of the aneurysm. During the sixth month of follow-up, the patient continued to do well without any symptoms or complications. However, 10 months after the operation, she developed dyspnea and was subsequently diagnosed with pneumonia. An aortic CT scan showed no change in the stent graft, and the reduced aneurysm had thrombosed. Treatment with antibiotics was ineffective, and her condition rapidly worsened. Twenty-four days after the admission for pneumonia, the patient passed away. Postmortem pathological analysis revealed miliary tuberculosis and histological findings were suggestive of tuberculous inflammation of the thrombosed aneurysm leading to right renal miliary tuberculosis (Fig 4). The family members of the patient agreed to allow us to publish her case details and images.

## Discussion

In the present case, we performed an emergency EVAR for a saccular aneurysm at the origin of the right renal artery with an aortoduodenal fistula, which caused hypovolemia and hemorrhagic shock. Pathological autopsy revealed accumulation of *Mycobacterium tuberculosis* in the cystic mass, which thrombosed and showed a tendency to shrink on the follow-up CT scans, leading to the diagnosis of a tuberculous mass. Infectious aortic aneurysms are reported^1^, ^2^, ^3^, ^4^, ^5^ to account for approximately 3% of all aortic aneurysms, and infectious aortic aneurysms caused by *M tuberculosis* are extremely rare. According to the report by Volini et al,^6^ there are two possible mechanisms for the development of tuberculous aortic aneurysms: (1) a direct invasion of the aortic wall from a tuberculous lesion such as in a lymph node or bone and an abscess around the aorta or (2) hematogenous spillover into the aortic feeding vessels or damaged aortic intima. It has been reported that more than 90% of the tuberculous infected masses have a saccular shape.^5^^,^^7^ In this case, the pathogenesis of the cystic mass was not clear because a prior infection was unknown, and the mass developed naturally in a healthy patient with no history of tuberculosis.

Although the patient was asymptomatic, the pathological autopsy findings revealed a lesion in the cystic mass caused by *M tuberculosis* that was not eradicated despite the mass undergoing thrombosis by stent grafting with the subsequent stopping of the bleeding. We expected that the chronic infection had rapidly progressed to miliary tuberculosis. Despite the increase in endovascular treatment in recent years, it must be chosen based on the urgency and the patient’s surgical tolerance. However, the cause of the infection may not be identified. Although there have been reports of good results with endovascular treatment of tuberculous aortic aneurysms,^1^, ^2^, ^3^, ^4^ the importance of antituberculosis drugs after an endovascular treatment has been highlighted. As for the surgical technique, in this case, reconstruction of the right and left renal arteries was performed using VIABAHN (covered stent) and a bare metal stent, in addition to the usual abdominal aortic stent graft. In recent years, there have been reports on the usefulness of the chimney and the sandwich techniques using covered stents and bare stents for pararenal aneurysms.^8^, ^9^, ^10^ Therefore, we believed that a right renal artery reconstruction with a covered stent was the only option for this saccular aneurysm at the origin of the right renal artery.

The operative time was short (61 minutes), and no endoleak was observed immediately after the operation. The progression of anemia owing to gastrointestinal bleeding had subsided, and thrombosis and reduction of the cystic mass were observed at an early stage. Therefore, we believed that this surgical technique was appropriate. Because aortoduodenal fistula had developed, the risk of postoperative stent graft infection was high, and thorough antibiotic therapy was considered essential and was administered in this patient. However, this strategy did not lead to a cure, the diagnosis was not confirmed until the patient’s condition progressed to miliary tuberculosis, and she died. We regret that we missed the timing of the open surgery and did not reach a definitive diagnosis as the patient’s progress after the endovascular treatment was good. As a future issue, there is the possibility that the cause of the aortic saccular aneurysm is a tuberculous infected aneurysm, although this is very rare. If a tuberculous infected aneurysm is suspected, postoperative antituberculosis therapy is imperative, even if the endovascular treatment is successful.

## Conclusions

In this study, we encountered a case of miliary tuberculosis 10 months after the operation, although the patient was brought to the emergency room with hemorrhagic shock owing to an aortoduodenal fistula and was saved by emergency EVAR. Pathological autopsy revealed a tuberculous, infected mass. Considering the possibility of tuberculosis as a cause of asymptomatic stent graft treatment of saccular masses and to consider preoperative examination, selection of antibiotics, and postoperative therapy including antituberculosis drugs is necessary in some cases.
